# Supplementary material for: A Data-Driven Approach to Link GC-MS and LC-MS with Sensory Attributes of Chicken Bouillon with Added Yeast-Derived Flavor Products in a Combined Prediction Model
Source: Metabolites. 2025 May 8;15(5):317. doi: 10.3390/metabo15050317 (PMC12113473; doi:10.3390/metabo15050317)
Supplement: Supplementary file 1 [file metabolites-15-00317-s001.zip › metabolites-3566920-supplementary.pdf]

Supplementary Table S1. Compositional factors of the chicken bouillon samples used in this study. Chicken bouillon samples consisted of chicken aroma mix (dosages: high, middle, low) and yeast product (YP). YP types are divided in four: process flavour, process flavour blend, yeast extract and yeast extract blend. The samples were assessed by panellists in different sessions, and a few samples were duplicated in the SBSE-GC-MS analysis. To prepare the chicken bouillon soups, an amount of bouillon was added to 1L of water.

Chicken bouillon samples				Sensory part	Analytical part
**No.**[1]	**Name**	**Chicken aroma dosage**	**Type of YP**	**Panel session**	**Technical duplicates**
1	CLPFA	low	process flavour	5	
2	CMPFA	middle	process flavour	3	
3	CLPFB	low	process flavour	6	
4	CMPFB	middle	process flavour	1	
5	CHPFC	high	process flavour	1	
6	CLPFC	low	process flavour	4	
7	CLPFD	low	process flavour	6	Yes
8	CMPFD	middle	process flavour	2, 4	Yes
9	CLPFE	low	process flavour	8	
10	CLPFF	low	process flavour	8	
11	CMPFF	middle	process flavour	4	
12	CHPFG	high	process flavour	8	
13	CLPFG	low	process flavour	2, 4	
14	CHPB1	high	process flavour blend	8	
15	CMPB1	middle	process flavour blend	7	
16	CHPB2	high	process flavour blend	2	
17	CMPB2	middle	process flavour blend	6	
18	CHPB3	high	process flavour blend	1	
19	CLPB3	low	process flavour blend	3, 5	
20	CHPB4	high	process flavour blend	8	Yes
21	CMPB4	middle	process flavour blend	1, 7	Yes
22	CHPB5	high	process flavour blend	4	
23	CMPB5	middle	process flavour blend	5	
24	CMPB6	middle	process flavour blend	6	
25	CLPB6	low	process flavour blend	3	
26	CHPB7	high	process flavour blend	5	
27	CLPB7	low	process flavour blend	1	
28	CMPB8	middle	process flavour blend	2	
29	CLPB8	low	process flavour blend	4	
30	CHPB9	high	process flavour blend	5	
31	CMPB9	middle	process flavour blend	8	
32	CHPB10	high	process flavour blend	3	
33	CMPB10	middle	process flavour blend	1	
34	CHPB11	high	process flavour blend	7	
35	CLPPB11	low	process flavour blend	3	
36	CMPB12	middle	process flavour blend	3	
37	CLPB12	low	process flavour blend	1	
38	CHPB13	high	process flavour blend	3	
39	CMPB13	middle	process flavour blend	4	
40	CMPB14	middle	process flavour blend	6	
41	CLPB14	low	process flavour blend	2	
42	CHYEA	high	yeast extract	6, 7	
43	CLYEA	low	yeast extract	2	
44	CHYEB	high	yeast extract	1	
45	CLYEB	low	yeast extract	7	
46	CHYEC	high	yeast extract	6	
47	CMYEC	middle	yeast extract	5	
48	CHYED	high	yeast extract	2	
49	CMYED	middle	yeast extract	3,5	
50	CMYEE	middle	yeast extract	8	
51	CLYEE	low	yeast extract	1	
52	CMYEF	middle	yeast extract	5	

53	CLYEF	low	yeast extract	1, 8	
54	CHYEG	high	yeast extract	3, 5	Yes
55	CLYEG	low	yeast extract	4	Yes
56	CMYEH	middle	yeast extract	8	
57	CLYEH	low	yeast extract	6	
58	CHYEI	high	yeast extract	6	
59	CLYEI	low	yeast extract	7	
60	CHYB1	high	yeast extract blend	6, 8	Yes
61	CMYB1	middle	yeast extract blend	2	Yes
62	CHYB2	high	yeast extract blend	2	
63	CMYB2	middle	yeast extract blend	7	
64	CHYB3	high	yeast extract blend	4	
65	CLYB3	low	yeast extract blend	3	
66	CHYB4	high	yeast extract blend	4	
67	CLYB4	low	yeast extract blend	7	
68	CMYB5	middle	yeast extract blend	7	
69	CLYB5	low	yeast extract blend	5	
70	CHYB6	high	yeast extract blend	3	
71	CMYB6	middle	yeast extract blend	2	

[1]: Chicken bouillon sample number

Supplementary Table S2. Gradient conditions of the LC-MS methods.

A) reverse phase chromatography

Time (min)	Flow rate (mL/min)	A (%)	B (%)
Initial	0.60	97	3
0.50	0.60	97	3
3.00	0.60	80	20
6.50	0.60	60	40
8.50	0.60	20	80
8.90	0.60	10	90
9.00	0.60	0	100
13.40	0.60	0	100
13.50	0.60	97	3
15.00	0.60	97	3

Mobile phase A: MilliQ water + 0.1% formic acid

Mobile phase B: Acetonitrile + 0.1% formic acid

B) HILIC Chromatography

Time (min)	Flow rate (mL/min)	A (%)	B (%)
Initial	0.50	100	0
1.20	0.50	100	0
9.16	0.50	25	75
14.00	0.50	25	75
14.20	0.50	100	0
18.00	0.50	100	0

Mobile phase A: 90% Acetonitrile + 10% water with 0.1% formic acid, ammonium formate

Mobile phase B: 10% Acetonitrile + 90% water with 0.1% formic acid, ammonium formate in water

Supplementary Table S3. XCMS parameter settings and feature filtering.

XCMS parameter settings		
**Parameter group**	**Parameter**	**Setting**
Peak integration	method	centWave
	ppm	10
	snthr	3
	peakwidth	5-10
	mzdiff	0.008
	prefilter peaks	3
	prefilter intensity	10
	noise	2
Retention time correction	method	obiwarp
	profStep	1
Grouping	method	density
	bw	5
	mzwid	0.008
	minfrac	0.1
	minsamp	2
CAMERA annotations	mzabs	0.002
	ppm	5
	sigma	6
	perfwhm	0.6

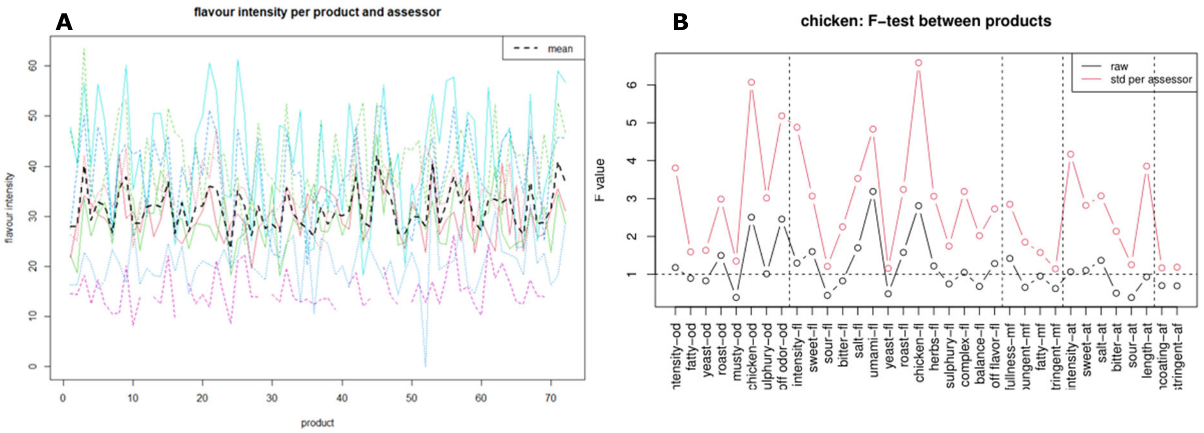

Supplementary Figure S1. (A) Scores for the Sensory attribute "flavour intensity" per bouillon sample given by each assessor (coloured lines) and the calculated mean value (discontinuous black line). **(B)** F-values per sensory attribute before (black) and after (red) applying standardization method (section 2.2).

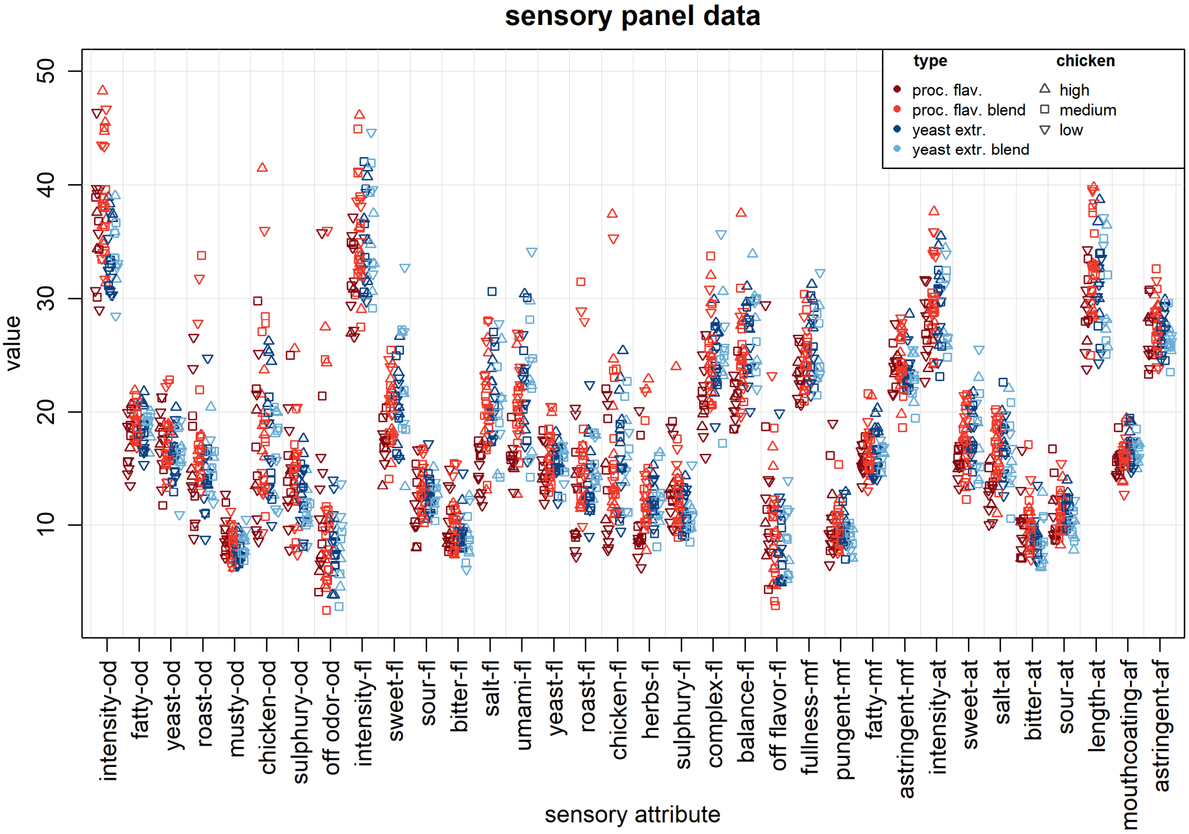

Supplementary Figure S2. Sensory panel data after standardisation, Scores for the Sensory attributes per assessor.

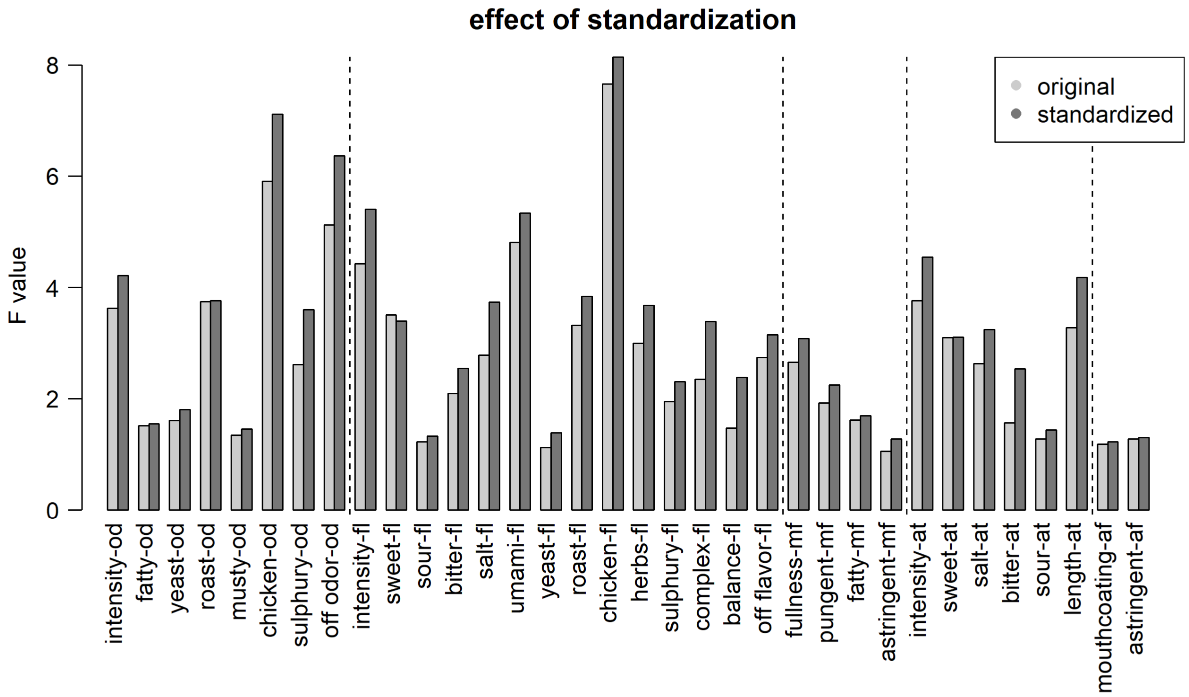

Supplementary Figure S3. Effect of standardization.

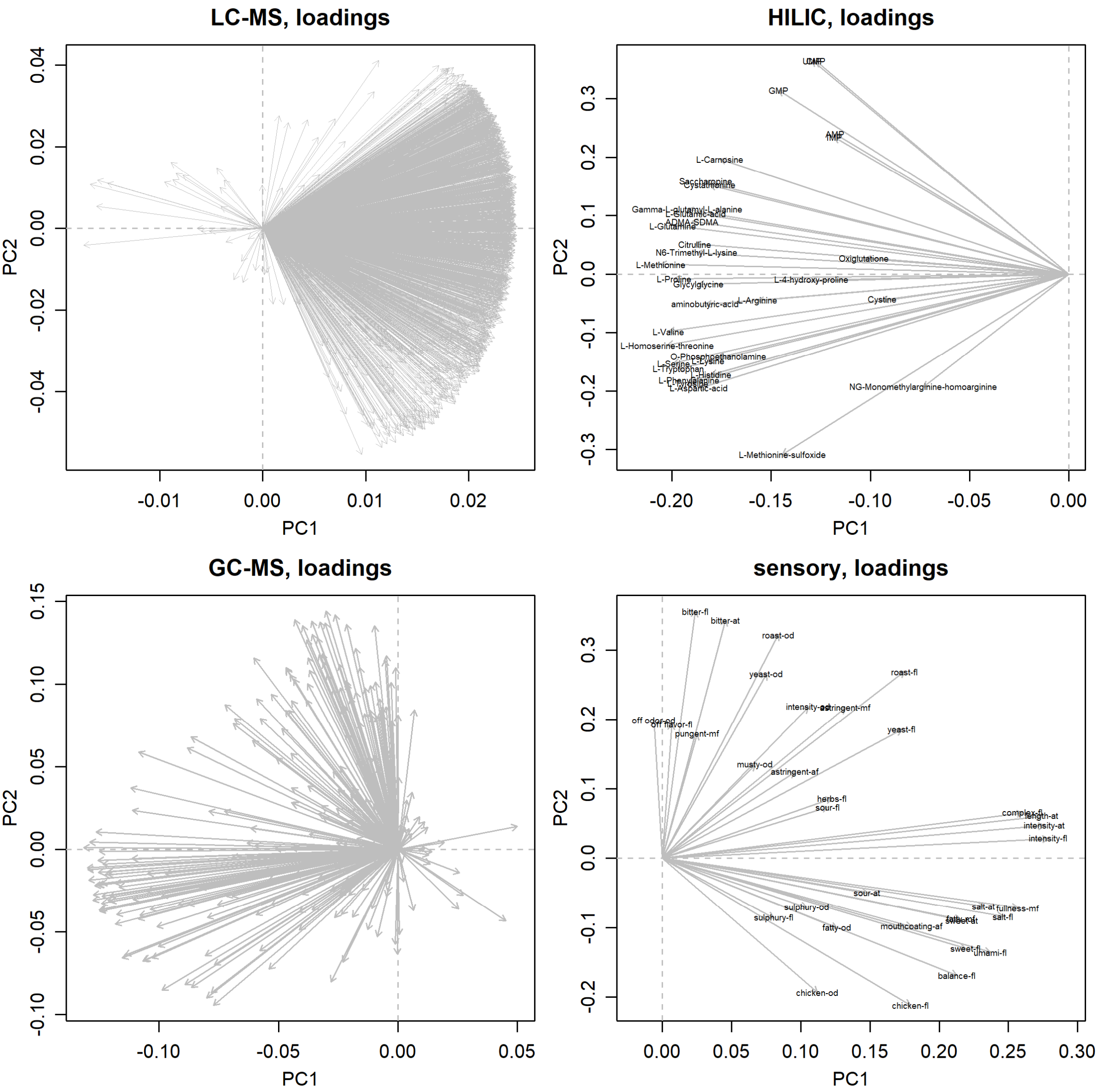

Supplementary Figure 4: Loadings of the PCAs shown in Figure 3.

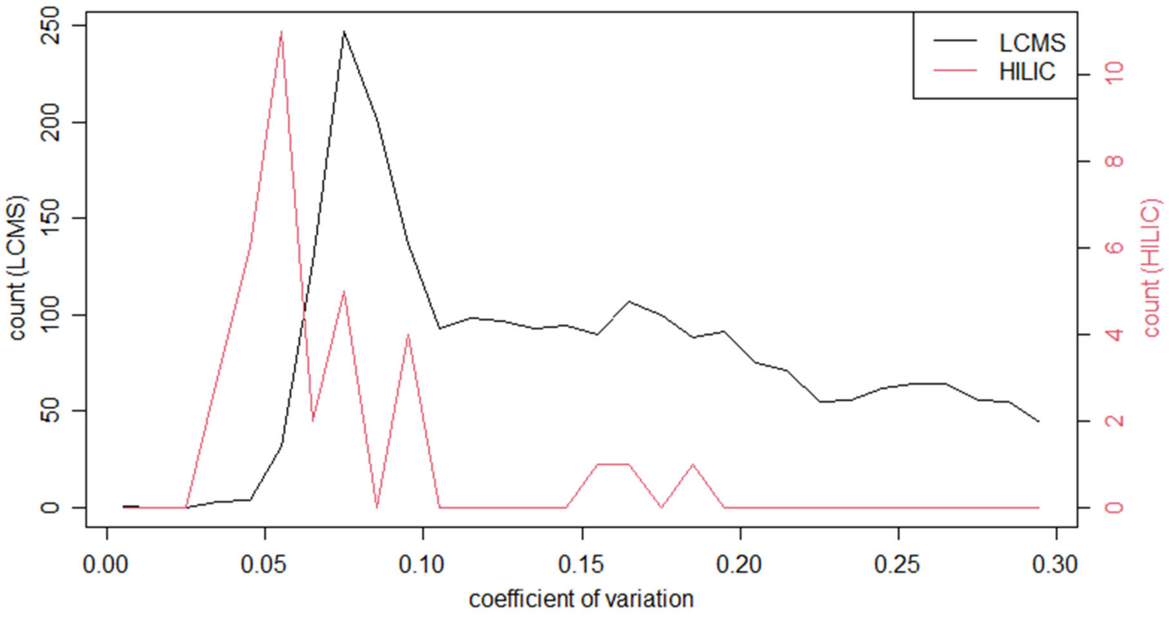

Supplementary Figure 5: Histogram of coefficients of variation of the quality control samples as a measure of precision for RPLC-MS and HILIC-MS

Supplementary Table 4. Putative identifications of the top highest ranked features measured by LC-MS, HILIC-MS and GC-MS of the sensory selected attribute models. The identification confidence levels of the annotated MS features are based on the levels 1-4 proposed by Sumner et al. and the feature's relevance for (other) MUVR-suggested min models is listed. Abbreviations: -af: afterfeel, -at: aftertaste, -mf: mouthfeel, -fl: flavor, -od: odor.

**Salt Flavor**				
**Feature**	**Measurement**	**Likely hit/formula**	**Id. conf. level**	**Relevance for sensory min. models**
H_GMP	HILIC	GMP	1	intensity-fl/at, sweet-fl/at, salt-fl/at, umami-fl, +5
H_IMP	HILIC	IMP	1	intensity-fl/at, sweet-fl/at, salt-fl/at, umami-fl, +3
P901	RPLC	N-gamma-L-Glutamyl-L-phenylalanine/Aspartame	2-3	salt-fl/at
P966	RPLC	C12H22N4O5	3	salt-fl/at
P816	RPLC	Aspartylphenylalanine/Phenylalanyl-aspartic acid	2-3	salt-fl
N1072	RPLC	C13H25N5NaO2P	3	salt-fl
H_UMP	HILIC	UMP	1	intensity-fl/at, sweet-fl, fatty-mf
N1031	RPLC	C16H19N0O5	4	sweet-fl

**Umami Flavor**				
**Feature**	**Measurement**	**Likely hit/formula**	**Id. conf. level**	**Relevance for sensory min. models**

H_GMP	HILIC	GMP	1	intensity-fl/at, sweet-fl/at, salt-fl/at, umami-fl, +5
H_IMP	HILIC	IMP	1	intensity-fl/at, sweet-fl/at, salt-fl/at, umami-fl, +3
N1535	RPLC	C18H36N4O4	4	umami-fl
H_CMP	HILIC	CMP	1	intensity-fl/at, sweet-fl/at, umami-fl, fullness-mf, +2
N578	RPLC	Leu-Ala-Ser, Ala-Val-Thr or Thr-Gly-Leu or isomer	2-3	umami-fl, sweet-at
H_UMP	HILIC	UMP	1	intensity-fl/at, sweet-fl, fatty-mf
N1809	RPLC	C19H27N3O6	4	umami-fl

**Sweet Flavor**				
**Feature**	**Measurement**	Likely hit/formula	**Id. conf. level**	**Relevance for sensory min. models**
H_GMP	HILIC	GMP	1	intensity-fl/at, sweet-fl/at, salt-fl/at, umami-fl, +5
N1470	RPLC	C17H23NNa2O5	4	sweet-fl
H_IMP	HILIC	IMP	1	intensity-fl/at, sweet-fl/at, salt-fl/at, umami-fl, +3
H_UMP	HILIC	UMP	1	intensity-fl/at, sweet-fl, fatty-mf
H_CMP	HILIC	CMP	1	intensity-fl/at, sweet-fl/at, umami-fl, +3
N2333	RPLC	C23H41N2O3PS	4	sweet-fl
N1031	RPLC	C16H19N0O5	4	sweet-fl
H_LGlutamic acid	HILIC	L.Glutamic.acid	1	intensity-fl, sweet-fl/at
N1589	RPLC	C15H25N3O8	4	sweet-fl
N1590	RPLC	C15H25N3O8	4	sweet-fl/ay
N851	RPLC	C16H19NNa2O3	4	sweet-fl
N1866	RPLC	C17H30N6O6	3	intensity-fl/at, sweet-fl, yeast-fl, complex-fl, length-at
SBSE19109	GC	homosalate	2	sweet-fl, bitter-fl, fatty-mf
N1415	RPLC	C19H27N3O4/C14H32N3NaO4S	3	sweet-fl, salt-at

P719	RPLC	Methionyl-Leucine or Isomer	2	sweet-fl
N2530	RPLC	C21H37N5O9	4	sweet-fl
P1838	RPLC	C17H30N4O5	3	sweet-fl
N1588	RPLC	non-identified	4	sweet-fl/at, sweet-at, salt-at

**Chicken Odor**				
**Feature**	**Measurement**	**Likely hit/formula**	**Id. conf. level**	**Relevance for sensory min. models**
H_Cystine	HILIC	Cystine	1	fatty-od, chicken-od/fl, sulphury-od/fl
SBSE7290	GC	2,4-nonadienal	2	fatty-od, chicken-od, bitter-fl
N2327	RPLC	N-Fructosyl glutamylphenylalanine or isomer	2	chicken-od, sulphury-od, chicken-fl
SBSE9017	GC	2,4-decadienal	2	fatty-od, chicken-od
SBSE3053	GC	1-octen-3-ol	2	chicken-od/fl, sulphury-od, sulphury-fl, sour-at
N303	RPLC	Traumatic acid or isomer	2-3	chicken-od
N1910	RPLC	C18H7N10NaO	3	chicken-od/fl, sulphury-od/fl, +2
SBSE9516	GC	2,4-decadienal	2	fatty-od, chicken-od
**Chicken Flavor**				
**Feature**	**Measurement**	**Likely hit/formula**	**Id. conf. level**	**Relevance for sensory min. models**
H_Cystine	HILIC	Cystine	1	fatty-od, chicken-od/fl, sulphury-od/fl
SBSE3053	GC	1-octen-3-ol	2	chicken-od/fl, sulphury-od/fl, sour-at

N1910	RPLC	$C_{18}H_{7}N_{10}NaO$	3	musty-od, chicken-od/fl, sulphury-od/fl, mouthcoating-af
N2639	RPLC	$C_{19}H_{38}N_{3}NaO_{8}S_{3}$	4	chicken-fl, fatty-mf
N2327	RPLC	N-Fructosyl glutamylphenylalanine or isomer	2	chicken-od/fl, sulphury-od
N1406	RPLC	non-identified	4	chicken-fl
N2803	RPLC	$C_{38}H_{50}N_{5}NaO_{3}S$	3	chicken-fl
N1243	RPLC	non-identified	4	chicken-fl
N2360	RPLC	$C_{21}H_{37}N_{2}O_{5}PS$	3	chicken-fl
N1238	RPLC	non-identified	4	chicken-fl
N1461	RPLC	$C_{15}H_{19}N_{8}NaO_{2}$	3	chicken-fl
N1253	RPLC	$C_{16}H_{21}N_{3}O_{6}$	3	chicken-fl
N973	RPLC	non-identified	4	chicken-fl

Roast Odor

Feature	Measurement	Likely hit/formula	Id. conf. level	Relevance for sensory min. models
SBSE11631	GC	$C_{14}H_{20}O_{2}$	3	roast-od
SBSE9895	GC	non-identified	4	roast-od, roast-fl
SBSE15025	GC	$C_{12}H_{11}N$	3	yeast-od, roast-od, bitter-fl/at
SBSE9846	GC	$C_{11}H_{18}N_{2}$	3	roast-od/fl
SBSE11886	GC	non-identified	4	roast-od
SBSE8385	GC	$C_{10}H_{16}N_{2}$	3	roast-od

SBSE3573	GC	C7H10N2	3	roast-od, bitter-fl, bitter-at
SBSE17608	GC	non-identified	4	yeast-od, bitter-fl, bitter-at
SBSE3473	GC	2-ethyl-5-methyl pyrazine	2	yeast-od, pungent-mf
N597	RPLC	Fructose-isoleucine, Fructose-leucine or isomer	2-3	yeast-od, roast-od/fl, bitter-fl, astringent-mf
SBSE11287	GC	2,3,5-trimethyl-6-isopentyl pyrazine	2	roast-fl
N2221	RPLC	non-identified	3	roast-fl

Roast Flavor

Feature	Measurement	Likely hit/formula	Id. conf. level	Relevance for sensory min. models
N597	RPLC	Fructose-isoleucine, Fructose-leucine or isomer	2-3	yeast-od, roast-od/fl, bitter-fl, astringent-mf
SBSE9895	GC	non-identified	4	roast-od/fl
SBSE14635	GC	4-methoxy-6-(2-propenyl)-1,3-benzodioxole	2	yeast-fl, roast-fl
N2221	RPLC	C22H15N4NaO5	3	roast-fl
SBSE6703	GC	4-terpineol	2	yeast-od, roast-fl
N256	RPLC	C9H15NO5	3	roast-fl
SBSE9846	GC	C11H18N2	3	roast-od/fl
SBSE132	GC	non-identified	4	roast-fl
N2175	RPLC	C20H7N3O5P2	4	roast-fl
SBSE7006	GC	terpineol	2	roast-fl
N2293	RPLC	C33H24N2	3	roast-fl
P506	RPLC	C11H21N3O2	3	roast-fl, astringent-mf

N2708	RPLC	non-identified	4	roast-fl
SBSE15145	GC	non-identified	4	roast-fl
P415	pos	L,L-Cyclo(leucylprolyl)	2-3	roast-fl
P428	pos	non-identified	3	yeast-fl, length-at
SBSE11287	GC	2,3,5-trimethyl-6-isopentyl pyrazine	2	roast-fl
